# Powers Obtained by Grimsby Corporation

**Published:** 1921-07-23

**Authors:** 


### NEW HEALTH LEGISLATION.

### Powers Obtained by Grimsby Corporation.

Many powers, decidedly novel in the way of health legislation, have been secured by the Grimsby Corporation in an Act which has recently passed through the House of Commons and the House of Lords.

Persons suffering from pulmonary tuberculosis, whose condition is infectious and whose home conditions do not provide facilities for precautions to prevent the spread of the disease, may be removed to hospitals. Application, however, must be made by the medical officer to the Courts before a consumptive may be so removed, and three days' notice must be given of an intention to seek such an order. The Corporation may financially aid dependent relatives.

In times of epidemics of infectious disease it will now be possible to search common lodging-houses and to examine medically the inmates.

To prevent the spread of disease in Sunday schools, places of entertainment, etc., it is forbidden to allow school children who have been excluded from elementary schools by reason of epidemics to attend such Sunday schools or places of amusement unless they first obtain certificates that such visits will be without undue risk of communicating disease to others. Persons who stop their employment at official request to prevent the spread of infectious disease can be compensated by the Corporation.

Dirty houses may be entered so that, where necessary, steps can be taken to secure their cleansing and fumigation. Wall-papers may be stripped and all other such measures taken to destroy vermin. Temporary shelter can meanwhile be provided by the Corporation for tenants, and those infected with vermin must submit to cleansing operations. Children suspected of being verminous can be inspected and compulsorily purified.

There is even legislation in regard to dustbins, which must be portable and of galvanised iron—no more of the old-fashioned ashpits or tubs being tolerated.

Careless or dirty tenants who are responsible for damaging closets or drains are brought under the scope of a penalty clause, and the Corporation may now make by-laws prescribing methods of flushing, etc., and for the prevention of blocking the drains therefrom.

In regard to food, the Corporation obtains powers to sample milk on the outskirts of the borough. It will be able to insist on cleanly and sanitary condition of premises where food is cooked or prepared. There is a requirement that meat or other articles of food shall be covered while being conveyed through the streets. Meat traders are forbidden to blow or inflate carcases, and a fine liability not exceeding £5 should be sufficient to put a speedy end to what is a disgusting practice, and one which is really unnecessary.

All new dwelling-houses must be provided with adequate food-storage accommodation, and there are powers to deal with existing houses where such storage is insufficient.

Various ice-cream clauses forbid its manufacture or storage in sleeping-rooms. Vendors must notify all cases of infectious disease.

Meat inspection by-laws are to be drastic, insisting on inspection before any meat is placed on sale—except foreign meat, which, of course, is first inspected abroad, and again at Smithfield or other such wholesale markets.

Infected persons are precluded from carrying on businesses associated with food. In cases of infectious disease the persons must tell the medical officer the address of any laundry to which clothing has been sent.

